# Additional record of *Tuponia* Reuter (Heteroptera, Miridae, Phylinae) from Korea, with a new synonym and discussion on distribution

**DOI:** 10.3897/BDJ.11.e104644

**Published:** 2023-05-18

**Authors:** Oh Min Suk, WonGun Kim, Jihwan Park, Seunghwan Lee

**Affiliations:** 1 Insect Biosystematics Laboratory, Department of Agricultural Biotechnology, Seoul National University, Seoul, Republic of Korea Insect Biosystematics Laboratory, Department of Agricultural Biotechnology, Seoul National University Seoul Republic of Korea; 2 Research Institute of Agricultural and Life Sciences, Seoul National University, Seoul, Republic of Korea Research Institute of Agricultural and Life Sciences, Seoul National University Seoul Republic of Korea; 3 207-404, Dogok Rexle Apt., Seoul, Republic of Korea 207-404, Dogok Rexle Apt. Seoul Republic of Korea; 4 Hanwha Ecometro 12, Nongogae-ro 10, Namdong-gu,, Incheon, Republic of Korea Hanwha Ecometro 12, Nongogae-ro 10, Namdong-gu, Incheon Republic of Korea

**Keywords:** Exaeretini, *Tuponia*, Korean Peninsula, new record, synonymy

## Abstract

**Background:**

The genus *Tuponia* Reuter, 1875 belongs to the subfamily Phylinae and comprises 91 species worldwide. Before this study, only *T.koreana* Kim & Jung had been recorded from the Korean Peninsula.

**New information:**

Two species of *Tuponia* Reuter, 1910 are recognised from the Korean Peninsula including the first record of *T.mongolica* Drapolyuk, 1980. *T.koreana* Kim & Jung, 2021 is proposed as a junior synonym of *T.chinensis* Zheng & Li, 1992. The species is identified, based on the dorsal habitus and male and female genitalic structures. A brief discussion of the distribution of Korean *Tuponia* species also is presented.

## Introduction

The phyline plant bug genus *Tuponia* Reuter, 1875 comprises 91 species worldwide (Schuh 2002, Kim et al. 2021). This group is distributed in the Palaearctic, Oriental Asia and northern Afrotropical Region and a large number of species use diverse *Tamarix* plants as their breeding host (Kerzhner and Josifov 1999, Li and Liu 2016). In the United States, *Tamarix* has been recognised as an invasive species, producing negative effects on ecosystems. For example, they accelerate salinisation of the soil (Ladenburger et al. 2006), increase wildfire severity by replacing fire-resistant plants and deplete groundwater (Pattison et al. 2011, Drus et al. 2013). In Korea, *Tamarix* has been planted on a limited basis as an ornamental tree, with natural populations only observed in Ansan and Incheon, which are distributed throughout the western coast of the Peninsula (NIBR 2011, Lee et al. 2018). Since a natural population exists in Korea and the plants can survive in many environments, research on related insects is important to understand more about the *Tamarix* species and its effect on the local ecosystem.

After the genus *Tuponia* was first erected by Reuter (1875), taxonomic analysis for this genus was actively conducted. In East Asia, Drapolyuk revised the subgenera *Chlorotuponia* Wagner, 1964 and *Tuponia* in Russia and Mongolia with the addition of 18 new species (Drapolyuk 1980, Drapolyuk 1982) and Chinese *Tuponia* was recently revised by Li and Liu (2016) with movement of 16 species including three new species. Later, Konstantinov (2016) reassessed the validity of some *Tuponia* species and synonymised three endemic Chinese species, including two of the new species described by Li and Liu (2016). In Korea, *Tuponiakoreana* Kim & Jung was recently described by Kim et al. (2021). Herein we discuss two species of *Tuponia* in Korea, including a new distributional record of *T.mongolica* Drapolyuk and suggest a new synonym of *Tuponiakoreana* Kim & Jung **syn. n.** with *T.chinensis* Zheng & Li. Images of the dorsal habitus and genitalic structures of both sexes were presented for these two Korean *Tuponia* species.

## Materials and methods

All examined specimens are deposited in the collection of Insect Biosystematics Laboratory, Research Institute for Agriculture and Life Science, Seoul National University, Korea (SNU) and National Institute of Biological Resources (NIBR), Incheon, Korea. External characteristics were observed under a Leica Z16 APO microscope and digital images were obtained with a Leica DMC 5400 camera. Genitalic structures were dissected and observed under a Leica DM 4000B microscope and images were taken using a digital camera combined with the microscope (Lumenera Infinity 3). All measurements (mean and range) are provided in millimetres, unless otherwise noted.

Terminology used to describe the genitalic structures follows Menard et al. (2014), Li and Liu (2016) and Kim et al. (2021), with the following abbreviations: Male: HP: hypophysis; SG: secondary gonopore; SL: sensory lobe. Female: DP: dorsal labiate plate; IL: interramal lobe; IS: interramal sclerite; RM: ramus; SR: sclerotized ring; VP: ventral labiate plate.

## Taxon treatments

### Tuponia

Reuter, 1875

726A4055-615A-515D-B8D9-30C1C1F362AF

Tuponia Reuter, 1875 - Reuter 1875: 98 as a subgenus of *Megalodactylus*, upgraded by Reuter 1878: 16.
Tuponia Capsus tamarisci Perris, 1857 Synonyms: TuponiacunealisReuter OM (1902) Capsidae novae mediterraneae. IV. Öfversigt af Finska Vetenskapssocietetens Förhandlingar B 44: 51–70.: 65. TuponianotatusFieber FX (1858) Criterien zur generischen Theilung der Phytocoriden (Capsini auct.). Wiener entomologische Monatschrift 2: 289–327, 329–347, 388, 1 pl.: 338.

#### Diagnosis

*Tuponia* can be recognised by the following characters: body elongate oval; dorsum somewhat shining, without distinct punctures and covered with pale, sericeous setae and dark setae; basic colouration greenish or yellowish-brown with brown, dark brown or reddish spots; membrane dark grey, vein pale green to brown; endosoma elongated, C-, S- or J-shaped; sometimes with sclerotised apical structures and membranous lobes; secondary gonopore usually developed between subapical part of sclerotised lobes; sclerotised ring elongated oval, interramal sclerite elongate and smooth. For detailed diagnostic characters and figures, see Drapolyuk (1980), Drapolyuk (1982), Li and Liu (2016) and Kim et al. (2021).

#### Distribution

Afrotropical Region, Oriental Region, Palaearctic Region (Drapolyuk 1980, Drapolyuk 1982, Kerzhner and Josifov 1999, Li and Liu 2016).

#### SubgenusTuponia

The subgenus can be recognised by the following characters: body elongate oval (male) or suboval (female), comparatively moderate to large (2.1–4.1 mm, usually around 3.0 mm); dorsum somewhat shining, without distinct punctures and covered with pale, sericeous setae; apical 1/3 of clavus and median part of hemelytra usually with suberect, dark brown to reddish setae, forms transverse line; basic colouration pale green to yellowish-green, partly brownish or tinged with red; membrane dark grey, vein pale green to brown; endosoma C-, S- or J-shaped, usually with one or two sclerotised apical structures; endosomal membrane rather developed, situated along apical processes; secondary gonopore usually developed between the subapical part of sclerotised lobes; female sclerotised ring elongate oval, surrounded by wide and weakly sclerotised labiate plates; bursa copulatrix with a pair of distinct, round structures dorsally; posterior wall with elongated, distally round interramal sclerites and weakly sclerotised, rough interramal lobe.

#### SubgenusChlorotuponia

This subgenus was established by Wagner (1964) and can be recognised by the following characters: body elongate oval (male) or suboval (female), comparatively small (1.7–2.5 mm, usually around 2.0 mm); dorsum somewhat shining, without distinct punctures and covered with long, pale hair and short, suberect brownish setae; basic colouration green to yellowish-green, usually concolorous; membrane dark grey, vein pale green to brown; endosoma C-, S- or J-shaped, usually with one or two sclerotised apical structures; endosomal membrane weakly developed, indistinct; secondary gonopore usually developed between the subapical part of sclerotised lobes; female sclerotised ring elongate oval, surrounded by wide and weakly sclerotised labiate plates; inner margin of bursa copulatrix with thin, arch-shaped sclerotised structure; posterior wall with elongated, distally round interramal sclerites and weakly sclerotised, interramal lobe with rough surface

### Tuponia (Chlorotuponia) chinensis

Zheng & Li, 1992

E6C1E63C-6EC6-5D76-B64D-737E1FE70C16

*Tuponiachinensis* Zheng & Li, 1992 - Zheng and Li 1992: 12.
*Tuponiakoreana* Kim & Jung, 2021 - *Kim et al. 2021*: 1268. **New synonymy.**

#### Materials

- **Type status:** Other material. **Occurrence:** recordedBy: Jihwan Park; individualCount: 10; sex: 5♂, 5♀; lifeStage: adult; **Taxon:** scientificName: Tuponia (Chlorotuponia) chinensis Zheng & Li, 1992; **Location:** country: Republic of Korea; stateProvince: Incheon-si; locality: Sorae Wetlands Ecology Park, Nonhyeon-dong, Namdong-gu; **Identification:** identifiedBy: MinSuk Oh; **Event:** eventDate: 31.vii.2021; habitat: on *Tamarixchinensis*; **Record Level:** institutionCode: SNU
- **Type status:** Other material. **Occurrence:** recordedBy: Jihwan Park; individualCount: 1; sex: 1♀; lifeStage: adult; **Taxon:** scientificName: Tuponia (Chlorotuponia) chinensis Zheng & Li, 1992; **Location:** country: Republic of Korea; stateProvince: Incheon-si; locality: Sorae Wetlands Ecology Park, Nonhyeon-dong, Namdong-gu; **Identification:** identifiedBy: MinSuk Oh; **Event:** eventDate: 31.vii.2021; habitat: on *Tamarixchinensis*; **Record Level:** institutionCode: NIBR

#### Diagnosis

**Figure 2.**
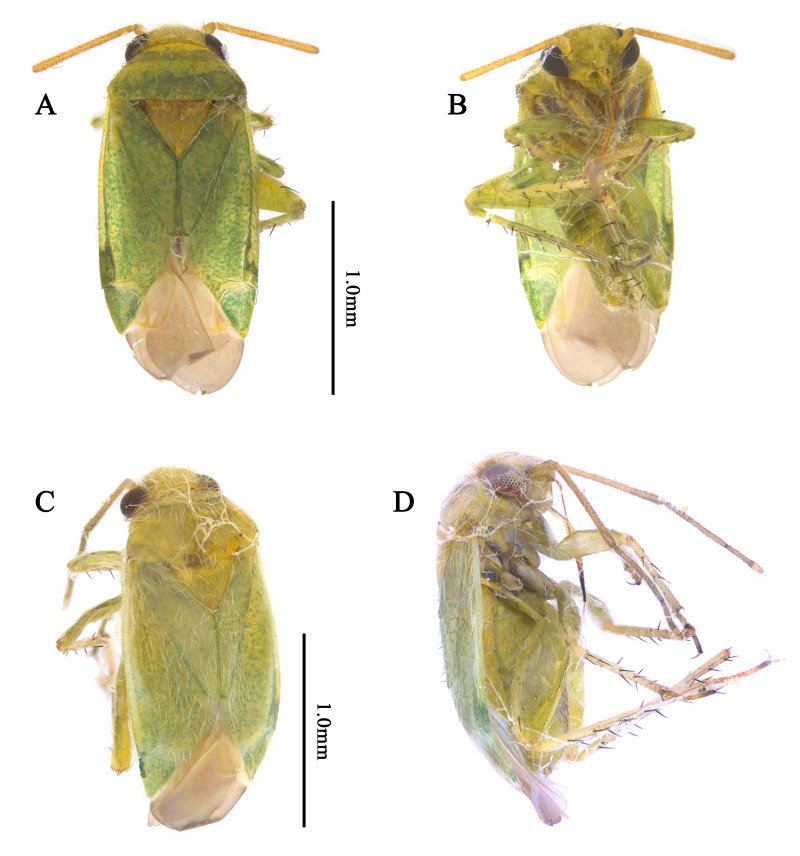
Dorsal habitus of Korean *Tuponiachinensis*. **A, B** Male; **C, D** Female. Scale bar: 1.0 mm.

Recognised by elongate oval body, 1.8–2.3 mm; basic colouration pale green to yellowish-green, weakly shining (Fig. 1A–C, Fig. 2A–D); dorsum covered with pale sericeous setae and dark brown setae; labium reaches metacoxa; hemelytra pale green, covered with long, sericeous setae and dark brown, simple setae; tibial spine blackish-brown, base without dark spot. Male genitalia (Fig. 4A–I): Endosoma S-shaped, with two elongated, twisted sclerites; secondary gonopore situated subapically between the two sclerites; left paramere with thick and round sensory lobe; sub-basal part of sensory lobe with elongated, pointed-end process; hypophysis sub-triangular, pointed-end; right paramere rather elongated and flat, slightly curved; hypophysis short. Female genitalia (Fig. 6A–C): Sclerotised ring ovoid, thin-rimmed; interramal sclerites slender and elongated. For detailed diagnostic characters and figures, see Zheng and Li (1992), Li and Liu (2016) and Kim et al. (2021).

##### Measurements

**Male** (n = 5). Total body length 1.86–2.07; head width across eyes 0.53–0.57; vertex width 0.29–0.32; lengths of antennal segment I–IV 0.14–0.18, 0.54–0.62, 0.38–0.39, 0.20–0.21; labial length 0.68–0.74; mesal pronotal length including collar 0.33–0.38; basal pronotal width 0.68–0.82; width across hemelytron 0.76–0.93; cuneal length 0.32–0.36; cuneal width 0.19–0.23; lengths of metafemur, tibia and tarsus 0.74–0.76, 1.07–1.14, 0.35–0.39. **Female** (n = 5). Total body length 1.85–2.06; head width across eyes 0.55–0.60; vertex width 0.32–0.35; lengths of antennal segment I–IV 0.15–0.19, 0.55–0.63, 0.34–0.41, 0.22–0.25; labial length 0.71–0.74; mesal pronotal length including collar 0.36–0.39; basal pronotal width 0.71–0.81; width across hemelytron 0.83–1.01; cuneal length 0.33–0.37; cuneal width 0.20–0.26; lengths of metafemur, tibia and tarsus 0.79–0.82, 1.08–1.18, 0.32–0.37.

#### Distribution

Korea (Incheon), China (Tianjin, Hebei, Shandong, Ningxia, Shaanxi) (Zheng and Li 1992, Li and Liu 2016, Kim et al. 2021).

#### Biology

Known host plant is *Tamarixchinensis* (Tamaricaceae) (Zheng and Li 1992, Li and Liu 2016, Kim et al. 2021).

#### Notes

We examined specimens of *Tuponiachinensis* in SNU and propose *T.koreana* Kim & Jung as a junior synonym of *T.chinensis* Zheng & Li. The diagnostic characteristics of *T.koreana* nearly match those of *T.chinensis* and the genitalia of the two nominal species are identical. Kim et al. (2021) separated *T.koreana* from *T.chinensis* by the following characters: i) metatarsal segment II distinctly shorter than segment III, ii) left paramere with one long process pointing down and iii) endosoma without visible secondary gonopore. However, as in Fig. 7, they only measured the length of each segment at the ventral side of the metatarsus and the actual length of segment II is not distinctly shorter than that of segment III. Additionally, in the structure of the left paramere, our specimen shows a straight lateral process (Fig. 4C). However, we could not find other structural differences and concluded that these were minor intraspecific variations. In addition, Kim et al. (2021) stated that a secondary gonopore of *T.koreana* is ‘clearly invisible,’ but it can be seen upon displacement of the two apical sclerites of the endosoma, as shown in Fig. 4E–H. When compared with the description of Zheng and Li (1992), the secondary gonopore of the Korean specimen looks conspecific in its subapical location and rugged margin.

### Tuponia (Tuponia) mongolica

Drapolyuk, 1980

1A125432-BF4C-5C1D-A052-FF25436C5EDA

*Tuponiamongolica* Drapolyuk, 1980 - Drapolyuk 1980: 63.
*Tuponiatamaricicola* Hsiao and Meng, 1963 - Hsiao and Meng 1963: 447, 449. (junior primary homonym of *Tuponiatamaricicola* Lindberg, 1939)
*Tuponiahsiaoi* Zheng and Li, 1992 - Zheng and Li 1992: 10.

#### Materials

- **Type status:** Other material. **Occurrence:** recordedBy: WonGun Kim; individualCount: 7; sex: 2♂, 5♀; lifeStage: adult; **Taxon:** scientificName: Tuponia (Tuponia) mongolica Drapolyuk, 1980; **Location:** country: Republic of Korea; stateProvince: Incheon-si; locality: Sorae Wetlands Ecology Park, Nonhyeon-dong, Namdong-gu; **Identification:** identifiedBy: MinSuk Oh; **Event:** eventDate: 19.viii.2022; habitat: on *Tamarixchinensis*; **Record Level:** institutionCode: SNU
- **Type status:** Other material. **Occurrence:** recordedBy: WonGun Kim; individualCount: 1; sex: 1♀; lifeStage: adult; **Taxon:** scientificName: Tuponia (Tuponia) mongolica Drapolyuk, 1980; **Location:** country: Republic of Korea; stateProvince: Incheon-si; locality: Sorae Wetlands Ecology Park, Nonhyeon-dong, Namdong-gu; **Identification:** identifiedBy: MinSuk Oh; **Event:** eventDate: 19.viii.2022; habitat: on *Tamarixchinensis*; **Record Level:** institutionCode: NIBR

#### Diagnosis

**Figure 3.**
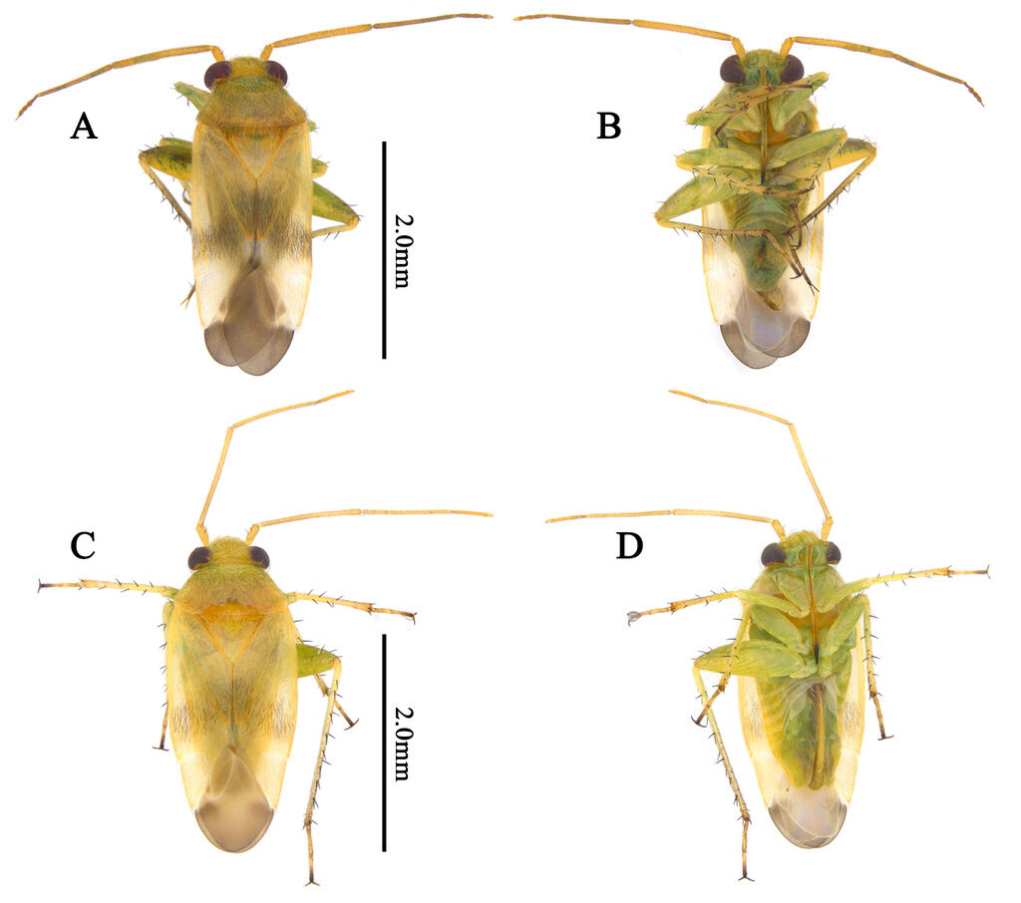
Dorsal habitus of Korean *Tuponiamongolica*. **A, B** Male; **C, D** Female. Scale bar: 2.0 mm.

**Figure 4.**
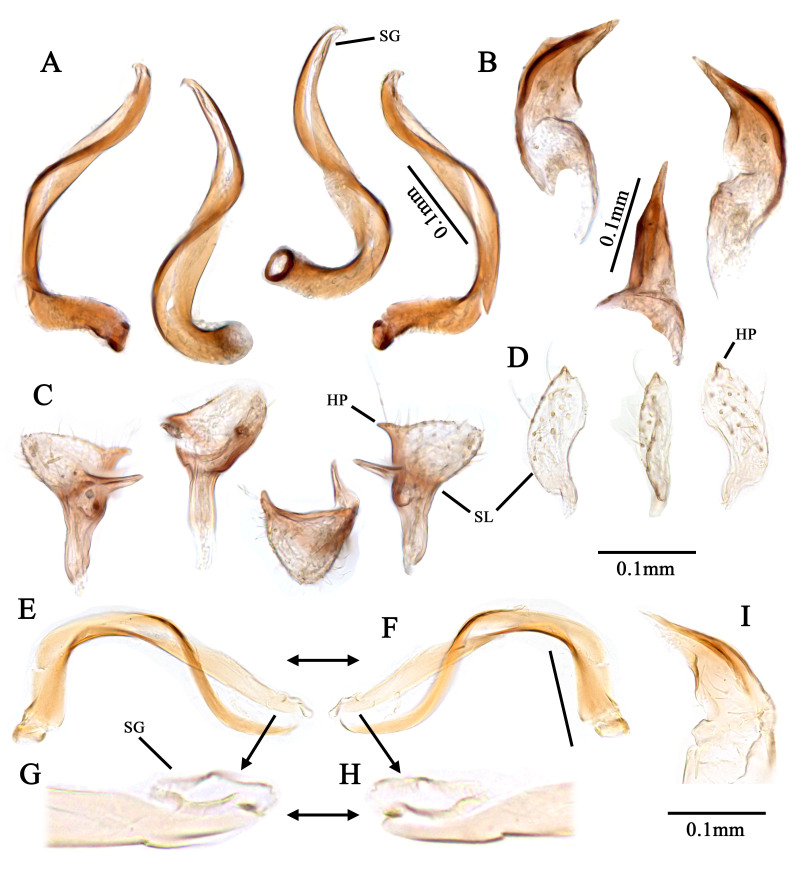
Male genital structure of Korean *Tuponiachinensis*. **A, E–H**, endosoma; **B, I** phallotheca; **C** left paramere; **D** right paramere. Scale bar: 0.1 mm.

**Figure 5.**
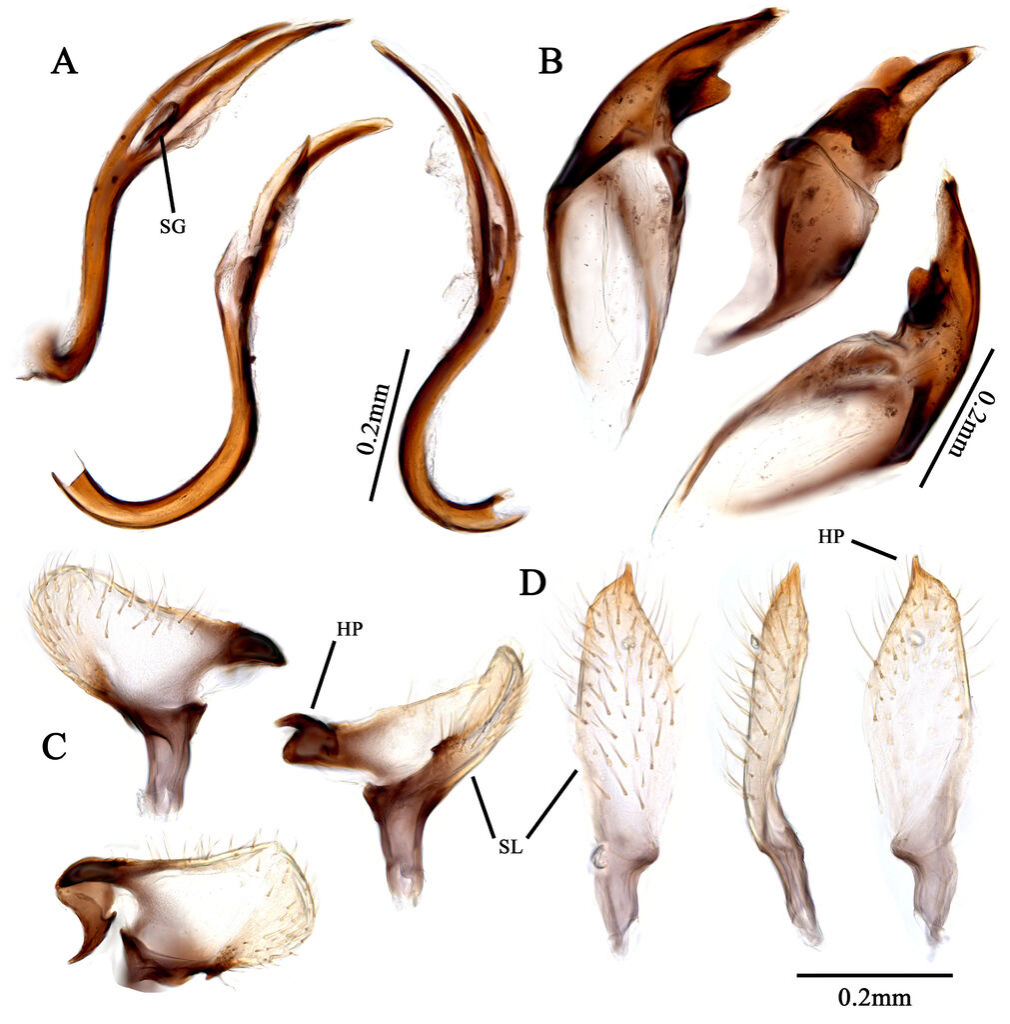
Male genital structure of Korean *Tuponiamongolica*. **A** endosoma; **B** phallotheca; **C** left paramere; **D** right paramere. Scale bar: 0.2 mm.

**Figure 6.**
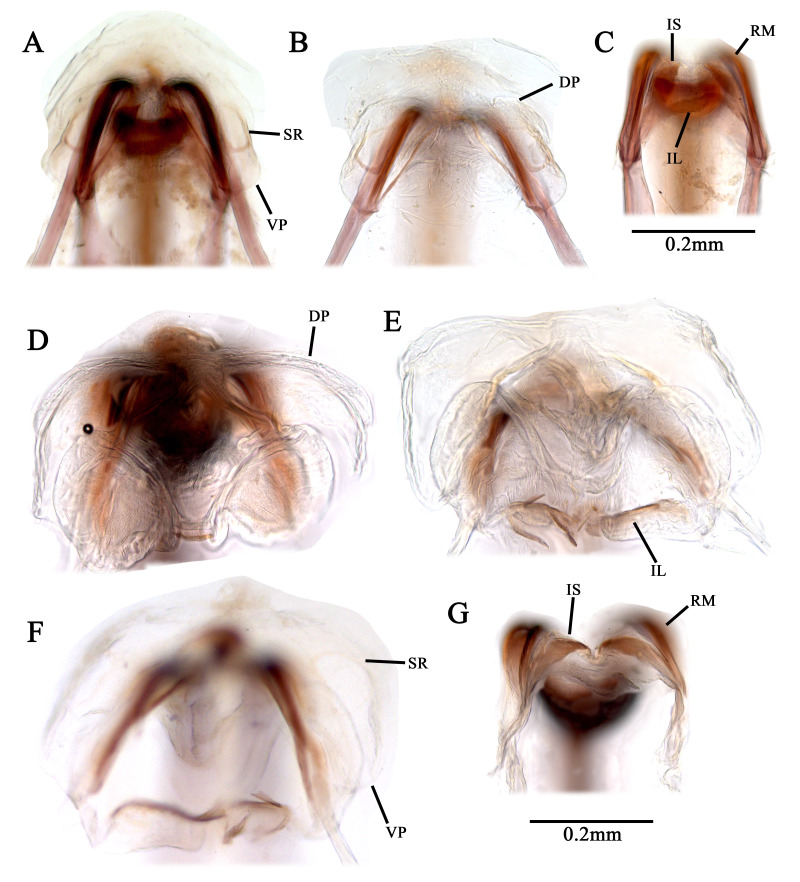
Female genital structure of Korean *Tuponia* species. **A–C**, *T.chinensis*; **D–G** *T.mongolica* (A, D: bursa copulatrix (before dissected posterior wall); **B, E** bursa copulatrix (dorsal view); **F** bursa copulatrix (ventral view); C, G: posterior wall). Scale bar: 0.2 mm.

**Figure 7.**
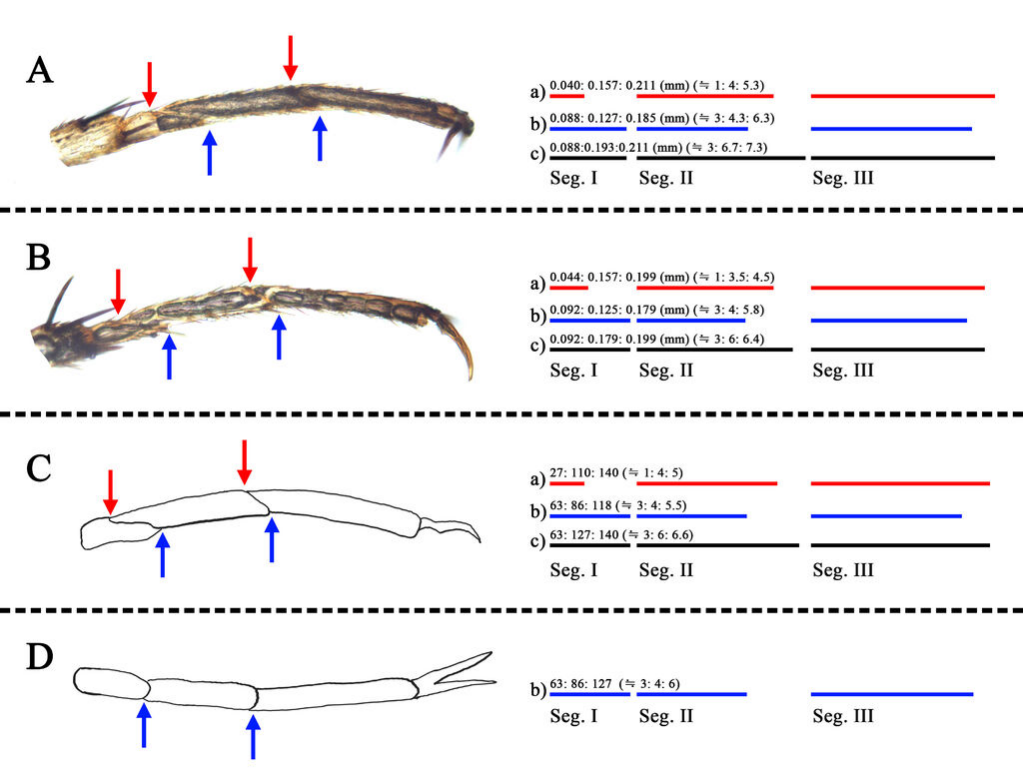
Metatarsus of *Tuponiachinensis*. Red arrow indicates segment borders in the dorsal side of tarsus and blue arrow indicates segment borders in the ventral side. Ratio of tarsal segments visualised with red bar (dorsal view: a), blue bar (ventral view: b) and black bar (actual length: c). **A** male; **B** female; **C** holotype male, a traced picture of Kim et al. (2021): fig. 1C; **D** a traced picture of Kim et al. (2021): fig. 1D.

Recognised by elongate oval body, 2.8–3.5 mm; basic colouration pale yellowish-green, weakly shining (Fig. 1D–F, Fig. 3A–D); dorsum covered with pale sericeous setae and dark brown setae; labium reaches metacoxa; hemelytra pale yellowish-green, partly tinged with pale orange; posterior half of clavus and posterior 1/3 of corium densely covered with brown setae; tibial spine blackish-brown. Male genitalia (Fig. 5A–D): Endosoma S-shaped, with two elongated, pointed-end sclerites and laterally serrate, membranous lobe; secondary gonopore developed between two sclerites; left paramere laterally wide; hypophysis twisted, apically hooked, sensory lobe with small pointed-end protuberance laterally. Female genitalia (Fig. 6D–G): Sclerotised ring ovoid, thin-rimmed; interramal sclerites slender and elongated. For detailed diagnostic characters and figures, see Drapolyuk (1980), Zheng and Li (1992) and Li and Liu (2016).

##### Measurements

**Male** (n = 2). Total body length 2.75–3.03; head width across eyes 0.76–0.79; vertex width 0.34–0.35; lengths of antennal segment I–IV 0.22, 0.90–0.99, 0.81, 0.36; labial length 1.15–1.16; mesal pronotal length including collar 0.54–0.55; basal pronotal width 0.99–1.07; width across hemelytron 1.09–1.16; cuneal length 0.47–0.54; cuneal width 0.28–0.29; lengths of metafemur, tibia and tarsus 1.02–1.09, 1.59–1.62, 0.50. **Female** (n = 5). Total body length 2.83–3.14; head width across eyes 0.75–0.80; vertex width 0.34–0.38; lengths of antennal segment I–IV 0.21–0.24, 0.97–1.00, 0.72–0.88, 0.33–0.38; labial length 1.08–1.23; mesal pronotal length including collar 0.50–0.55; basal pronotal width 1.07–1.14; width across hemelytron 1.23–1.29; cuneal length 0.53–0.57; cuneal width 0.29–0.32; lengths of metafemur, tibia and tarsus 1.04–1.16, 1.61–1.67, 0.50–0.56.

#### Distribution

Korea (Incheon), China (inner Mongolia, Shandong, Hebei, Ningxia), Mongolia (Drapolyuk 1980, Li and Liu 2016).

#### Biology

Known host plants are *Tamarix* sp. and *Tamarixchinensis* (Tamaricaceae) (Drapolyuk 1980).

#### Notes

This species can be confused with *T.jaxartensis* Drapolyuk and *T.zhenyuanensis* Li & Liu, from which it is easily distinguished by endosoma with laterally serrate and elongated apical sclerites, phallotheca with a fin-like protrusion at the inner margin and a structural difference of parameres.

## Discussion

### Distributions of Korean Tamarix population and Tuponia

In this work, *T.chinensis* Zheng & Li and *T.mongolica* Drapolyuk were found at a coastal wetland near Incheon. This area provides an adequate environment for *Tamarix* and is adjacent to an international Airport, which may have been its source of introduction. According to a recent study, it is assumed that Ansan and Incheon populations of *Tamarix* were introduced about 40 years ago from China (Beijing) and from another unknown origin (Lee et al. 2018). Kim et al. (2021) also mentioned this hypothesis and suggested the need for subsequent research on the distributions of *T.koreana* and the closely allied species *T.chinensis*. Since these two species are regarded as conspecific, we can assume that *T.chinensis* was introduced along with the *Tamarix*. To support this, a comparison of the relationships of *Tamarix* in naturalised and intentional ornamental populations is crucial. In addition, considering the *Tuponia* diversity of China (Li and Liu 2016), further investigations may yield additional records for Korean *Tuponia*.

## Supplementary Material

XML Treatment for Tuponia

XML Treatment for Tuponia (Chlorotuponia) chinensis

XML Treatment for Tuponia (Tuponia) mongolica

## Figures and Tables

**Figure 1a.**
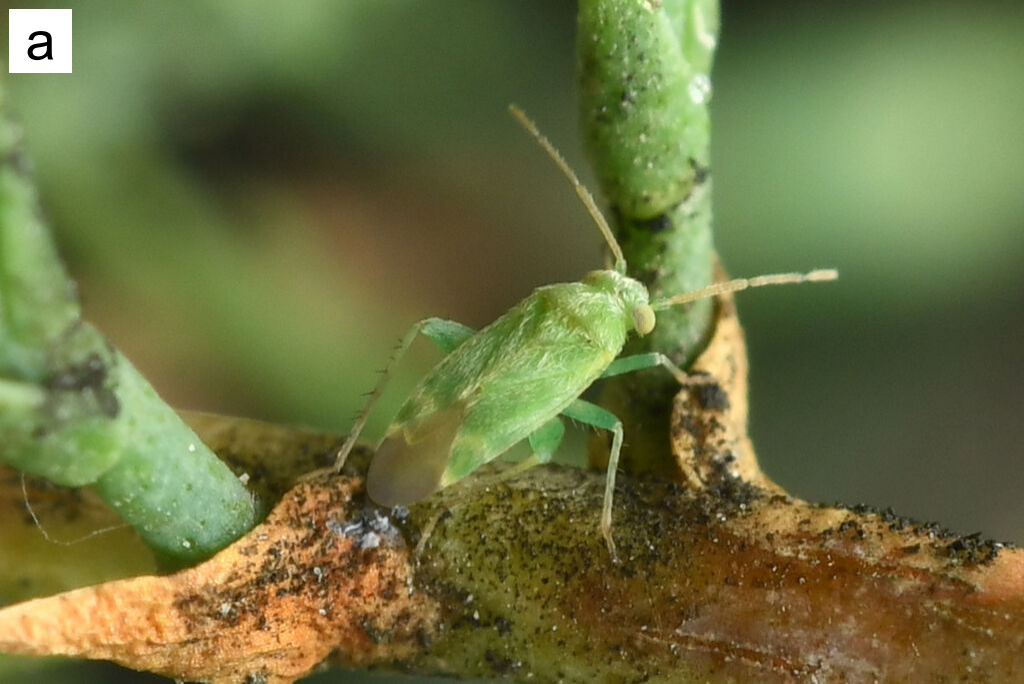
A: *Tuponiachinensis*, male;

**Figure 1b.**
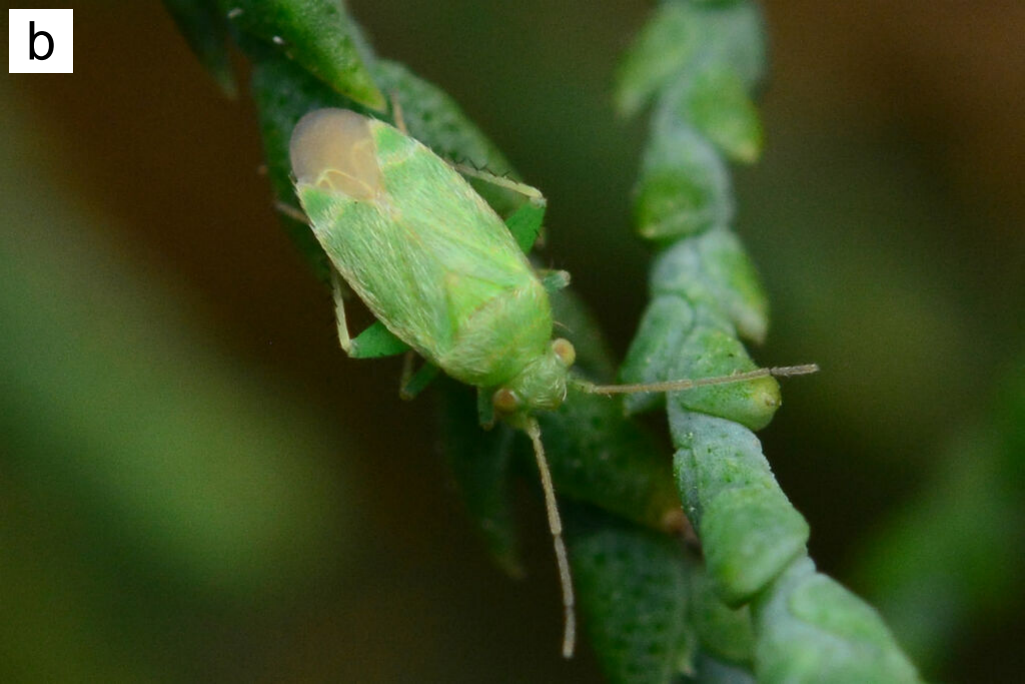
B: ditto, female;

**Figure 1c.**
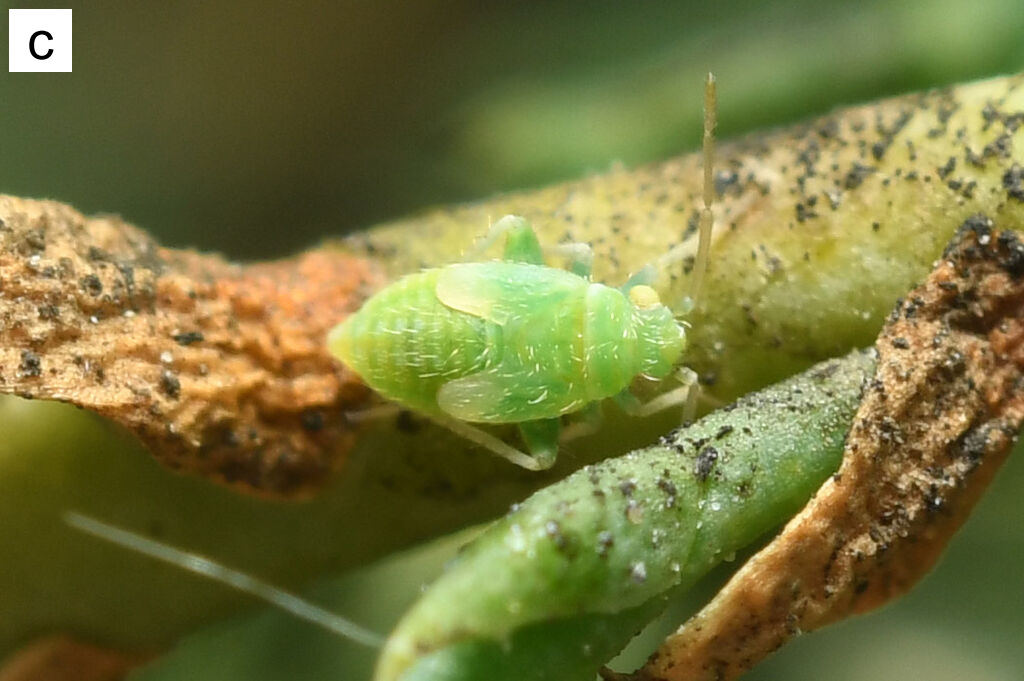
C: ditto, last instar;

**Figure 1d.**
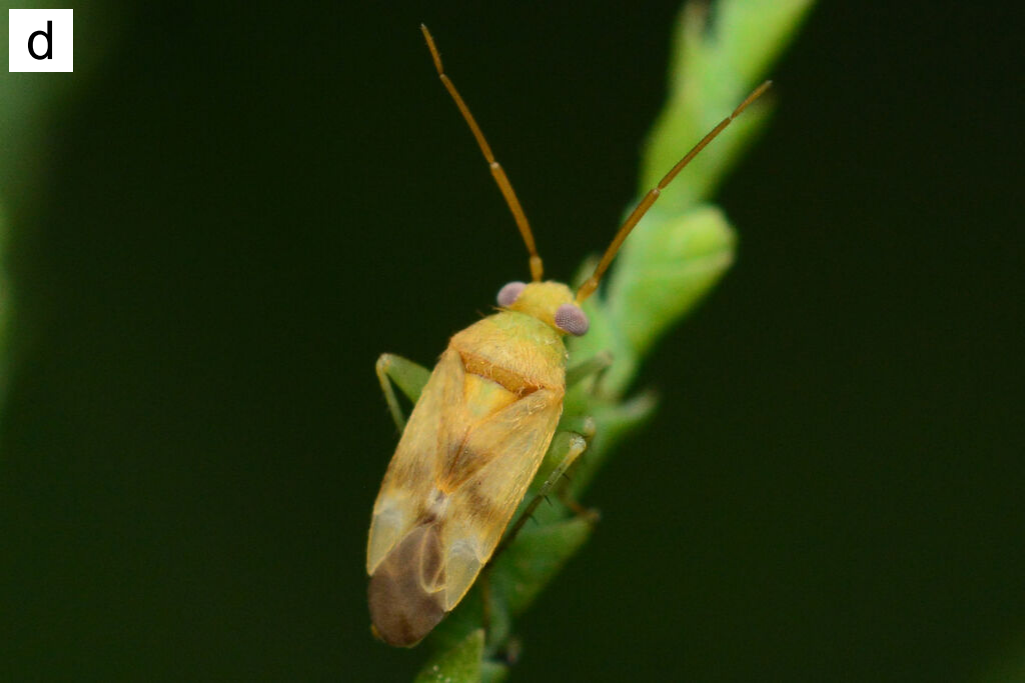
D: *T.mongolica*, male;

**Figure 1e.**
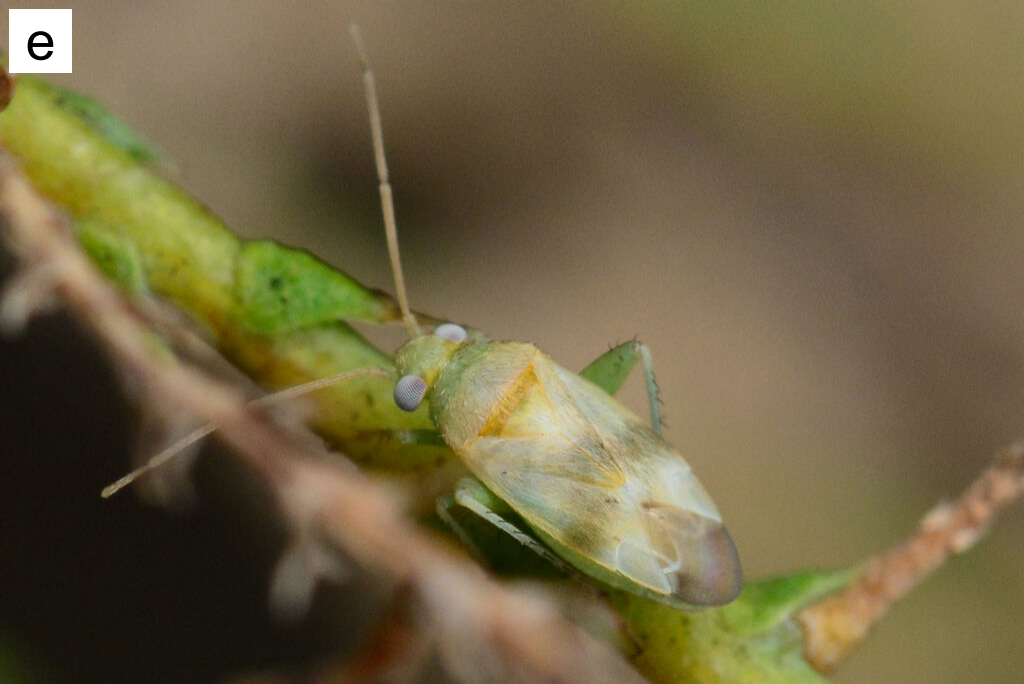
E: ditto, female;

**Figure 1f.**
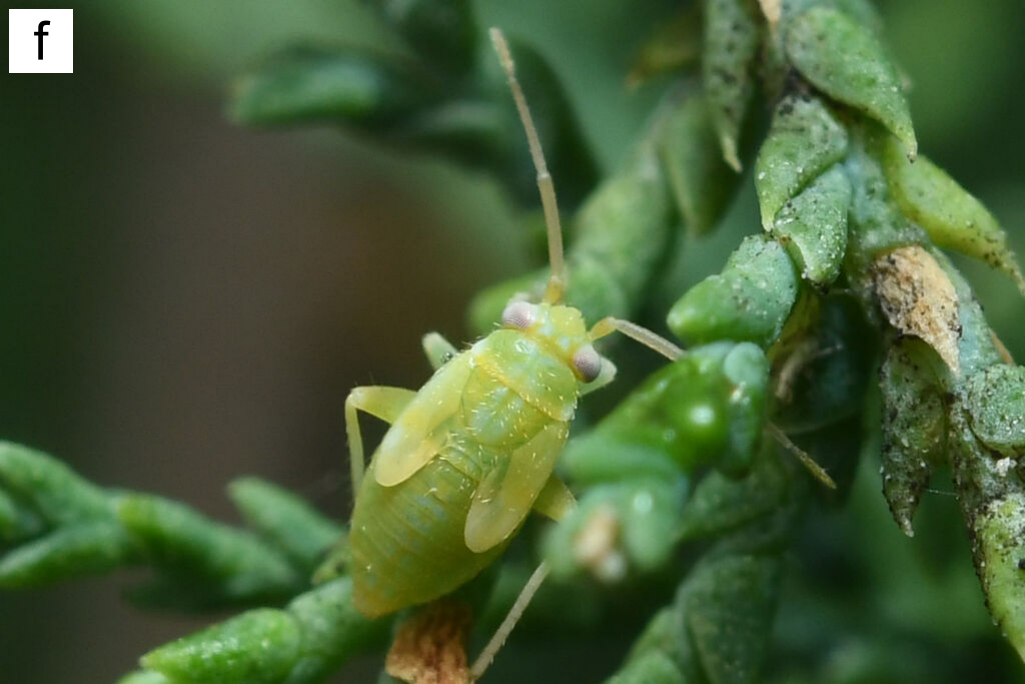
F: ditto, last instar.
